# Epidemiology of episodic adenolymphangitis: a longitudinal prospective surveillance among a rural community endemic for bancroftian filariasis in coastal Orissa, India

**DOI:** 10.1186/1471-2458-5-50

**Published:** 2005-05-19

**Authors:** Bontha V Babu, Abhay N Nayak, Kalpataru Dhal

**Affiliations:** 1 Division of Epidemiology, Regional Medical Research Centre, Indian Council of Medical Research, Bhubaneswar – 751 023, India

## Abstract

**Background:**

The epidemiological knowledge on acute condition of lymphatic filariasis is essential to understand the burden and issues on management of the disease.

**Methods:**

A one year long longitudinal prospective surveillance of acute adenolymphangitis (ADL) was carried out in rural population of Orissa, India.

**Results:**

The annual incidence of ADL per 1000 individuals is 85.0, and is slightly higher (*P *> 0.05) in male (92.0) than in female (77.6). A steady rise in the incidence of ADL episodes along with the age is recorded. The distribution indicates that persons with chronic disease are more prone to ADL attacks. The average number of episodes per year is 1.57 (1.15 SD) per affected person, and is gender dependent. Duration of the episode varies from 1 to 11 days with mean duration of 3.93 (1.94 SD) days. The chronic disease is the significant predictor for the duration of the episode. The data show that fever and swelling at inguinal regions are most common symptoms.

**Conclusion:**

The incidence, frequency and duration of ADL episodes in this community are similar to that of other endemic areas. As the loss due to these ADL episodes is substantial, it should be considered while further estimating the burden due to lymphatic filariasis. The disability and loss caused by chronic forms of filariasis is higher, and the additional incapacity caused by the ADL episode, majority of which occur among chronic filariasis patients, further poses the burden on individuals and their families. Hence, morbidity management measures to prevent ADL episodes among endemic communities are to be implemented.

## Background

Lymphatic filariasis (LF) is associated with a wide range of clinical signs, symptoms and sequelae, which are influenced by a variety of factors related to host and parasite. Acute episodes of adenolymphangitis (ADL) is one of the symptoms and this acute clinical manifestation is characterised by recurrent attacks of fever associated with inflammation of the lymph nodes and or lymph vessels [1]. The importance of acute clinical manifestations, i.e. ADL, in natural progression of the disease, particularly the development of chronic disease has been recognised by filarialogists [2-4]. Though the need of systematic epidemiological studies on acute LF or ADL is recognised, a few studies have been undertaken in different endemic areas [5-8]. The epidemiological information is useful to estimate the burden of the disease and to develop morbidity management strategies. The present paper, based on a longitudinal prospective fortnightly surveillance, reports some epidemiological aspects of ADL episodes among rural communities of Orissa, India, which is endemic for LF caused by *Wuchereria bancrofti*.

## Methods

### Study area

This one year longitudinal prospective surveillance for acute ADL episodes was carried out in two villages in Khurda district of Orissa, India. These two villages are close together by less than two kilometres and the geographical coordinates of these villages are 20° 11' N and 85° 40' E, and 20° 10' N and 85° 38' E. The study area is known for its endemicity for LF caused by *Wuchereria bancrofti*, which is transmitted by *Culex quinquefasciatus*. The microfilaria rate and density are 9.4% and 769 mf/ml respectively [9]. The total disease and chronic disease rates are 12.5% and 7% respectively [10]. The prevalence of infection based on circulating filarial antigenaemia is 39.9% [11]. One round of mass drug administration of diethylcarbamazine was given in the district in 1997 and recorded around 30% of treatment compliance (BV Babu, unpublished data). However, no data specific to these two villages are available. The area is rural and its inhabitants are mostly small farmers and daily wage labourers.

### Data collection

A population of 1329 (685 males and 644 females) of two villages were monitored for one year, during March 2000 – February 2001. Initially, the census of the villages was conducted for demographic information and identities of all individuals. The investigators visited all the households for every fortnight to detect individuals who were suffering or had suffered from an acute attack during that fortnight. In the present study, an acute ADL episode was defined as the presence of local signs and symptoms such as pain, tenderness, local swelling and warmth in the groin, with or without associated constitutional symptoms such as fever, nausea and vomiting [12]. The investigators, along with paramedical staff explained in the local language the symptoms of acute episodes, which are common in this endemic population. As there are no other diagnostic tools to identify ADL episodes, the present method of symptomatic diagnosis was used. This method of diagnosis through local terminologies was found to be highly specific (specificity = 0.980) and sensitive (sensitivity = 0.978) for diagnosing ADL [5]. For individuals identified as affected with acute attacks during last fortnight, the details including clinical symptoms, duration, etc. were recorded, by recall method and in case of ongoing episodes during visits, they were monitored. Majority of these cases are examined during the episode by physicians of local health institution. The available medical records/prescriptions were used as adjunct. A few episodes are clustered in members of the same household. The affected individuals were tested for microfilaraemia. A finger-prick thick blood film was prepared, using blood collected after 10 o'clock night, stained with Leishman's stain and entire film was examined for microfilariae.

### Data analysis

The data were analysed through SPSS V.8 for Windows. The variation in incidence, frequency, number and duration of ADL episodes were assessed by employing the following statistical tests. Standardised normal Z-tests were performed to assess the variation in the incidence between different groups. Differences in number of episodes and duration of episodes were assessed by t-tests and analysis of variance (ANOVA). Multivariate ANOVA by regression was used to examine the effect of gender, age and pathological condition on the number and duration of episodes in affected individuals. The seasonal variation in the frequency of ADL episodes was assessed by χ^2 ^test.

## Results

### Incidence and distribution of acute episodes

**Figure 1:**
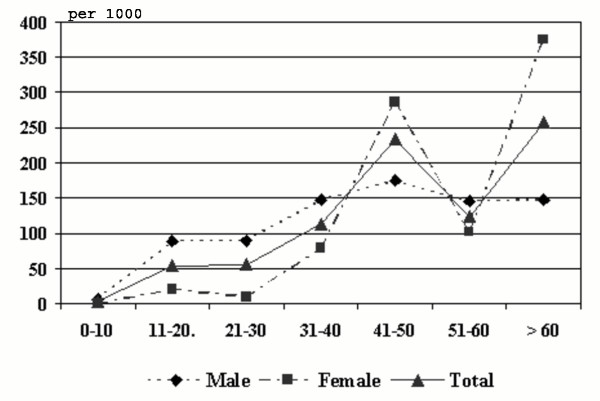
Relation between annual incidence of ADL episodes and different age groups among male, female and total population.

Number of fortnightly rounds completed during one year of study is 25 and 113 episodes were encountered among 72 patients in both villages. The annual incidence per 1000 population is 92.0 and 77.6 among men and women respectively, and the overall incidence is 85.0 per 1000 population. The difference between male and female is not significant (*P *> 0.05). However, the annual incidence is significantly high (*P *< 0.001) among individuals with chronic disease (957.0) than those without any chronic manifestations (19.4). The age-wise incidence of ADL episodes indicates a steady rise in the incidence of episodes along with age among males and females, except in the age group of 51–60 years (Fig. 1).

A total of 72 (52 males and 20 females) individuals of the study population (5.42%) were affected with acute ADLs. The age of the affected individuals varies from 7 years to 75 years with mean age of 36.23 ± 16.46 (SD) in case of male and 45.30 ± 12.15 (SD) in case of female, with a significant difference between two genders (*P *< 0.05). Among the total 72 patients suffered from acute episodes, overt chronic symptoms were found among 53 patients (73.61%). Out of total 113 episodes, 47 (41.59%) episodes are associated with lymphoedema, 38 (33.63%) with hydrocele, 4 (3.54%) with both lymphoedema and hydrocele and 24 episodes (21.24%) in individuals without chronic manifestations (Table 1). The mean age of patients with chronic manifestations (40.11 ± 15.22 SD) is slightly higher than individuals without any chronic manifestations (34.95 ± 17.68 SD), but the difference is not significant (*P *> 0.05).

**Table 1:** Association of gender, pathology group and age group with mean number of episodes and duration of episodes and details of multivariate ANOVA.

Independent Variable	Total number of patients in each category	Total number of episodes in each category	Mean number of episodes		Mean duration (days) of episode	
			
			Mean ± SD	Coefficient (*P*)^a^	Mean ± SD	Coefficient (*P*)^b^
Gender						
Male	52	63	1.21 ± 0.57	1.169 (0.000)	3.95 ± 2.05	0.508 (0.221)
Female	20	50	2.50 ± 1.67		3.90 ± 1.79	
Pathology group						
Hydrocele	31	38	1.23 ± 0.67	0.276 (0.071)	4.56 ± 2.34	0.505 (0.034)
Lymphoedema	19	47	2.47 ± 1.71		3.74 ± 1.76	
Both	3	4	1.33 ± 0.58		3.67 ± 1.15	
None	19	24	1.26 ± 0.56		3.52 ± 1.62	
						
Age group						
7–20 years	10	16	1.60 ± 1.08	0.002 (0.768)	4.38 ± 1.82	0.023 (0.0557)
21–40 years	30	40	1.33 ± 0.84		4.24 ± 2.41	
41–60 years	24	42	1.75 ± 1.45		3.60 ± 1.51	
61–75 years	8	15	1.88 ± 1.25		3.64 ± 1.80	
Total	72	113	1.57 ± 1.15		3.93 ± 1.94	

^a^For number of episodes: *F *= 9.317 (*P *< 0.001), Adjusted R^2 ^= 0.260

^b^For duration of episodes: *F *= 2.607 (*P *< 0.05), Adjusted R^2 ^= 0.041

### Frequency and duration of acute episodes

As 113 episodes were recorded among 72 patients, 20 (27.8%) patients experienced more than one episode. Majority of patients (52; 72.2%) experienced acute episode only once in the year. It is followed by 12.5% of patients with two episodes, 6.9% of patients with 3 episodes, 5.6% of patients with 4 episodes, and 1.4% of patients each with 5 and 7 episodes per year. Among the individuals with multiple episodes, majority (16 out of 20) are with chronic filarial symptoms (P < 0.001). The average number of episodes per year is 1.57 (1.15 SD) per affected individual. The multivariate ANOVA employed to assess the influence of age, gender and pathology on number of episodes per affected persons indicated that only gender is identified as significant predictor to the number of episodes per affected individual (P < 0.001) (Table 1). Similar analysis is carried out to assess the effect of age among hydrocele patients, and age and gender among lymphoedema patients. These variables have no impact on frequency of episodes among hydrocele and lymphoedema patients (P > 0.05). There is significant variation in the frequency of ADL episodes with seasons of the year (P < 0.001). The average numbers of episodes per month during summer (March to June), rainy season (July to October) and winter (November to February) are 11.00, 10.75 and 6.50 respectively.

Duration of the episode varies from 1 day to 11 days and the mean duration is 3.93 days (1.94 SD). In majority of cases, the episodes persisted for 3 days (26.4%), followed by 4 days (22.2%), 2 days (15.3%), 8 days (9.7%), 5 days (8.3%), 1 day (5.6%), 6 days (5.6%), 7 days (5.6%) and 11 days (1.4%). When individuals' age, gender and pathology were considered simultaneously in multivariate ANOVA, the individuals' pathological condition emerged as a significant predictor to the mean duration of episode (*P *< 0.05) (Table 1). When the hydrocele and lymphoedema patients are considered separately, age in hydrocele patients and, age and gender in lymphoedema patients have no significant impact on the duration of episode (*P *> 0.05).

### Clinical symptoms associated with ADL episodes

Distribution of various clinical symptoms among these acute cases is presented in Table 2. Occurrence of fever (77.87%) and swelling of inguinal regions (63.72%) are most common symptoms of acute cases. In the local language, swelling of lymphatic nodes is known as *Bagi *or *Pichhuli*, and is commonly perceived as the initial symptom of LF among the study population. Pain and tenderness in different body parts are seen in majority of cases. Presence of local swelling, anorexia, nausea and vomiting are associated with ADL among majority of cases. It is attempted to examine the differences in clinical presentation across gender and pathological groups. There are no significant variations in occurrence of these symptoms except in occurrence of pain and tenderness. Pain and tenderness in genitals is common in hydrocele patients, and hence more frequent in male patients. In lymphoedema patients, pain and tenderness are noticed in lower limbs. No association was found between occurrence of these symptoms and age groups. The microfilarae data indicated that only 8, out of 72 acute patients (11.11%) are microfilaraemic.

**Table 2:** Distribution of associated symptoms among the ADL cases.

Symptom	Prevalence among episodes	Percentage
Pain in		
Upper arms	12	10.62
Legs	83	73.45
Genitals	29	20.00
Breasts	1	0.88
Tenderness in		
Upper arms	10	8.85
Legs	13	11.50
Genitals	24	21.24
Lymphangitis in		
Left inguinal region	34	30.09
Right inguinal region	38	33.63
Left papillary	3	2.65
Right papillary	3	2.65
Swelling	92	81.42
Fever		
High	61	53.98
Low	27	23.89
Anorexia	72	63.72
Nausea	61	53.98
Vomiting	32	28.32
Microfilaraemiae	13	11.50^a^

^a^percentage is to total number of patients

## Discussion

To develop strategies for relieving and preventing the suffering of filarial patients, it is essential to understand the epidemiology of various forms of morbidity. The information on acute form of LF, particularly on ADL is sporadic from a few endemic regions. The present longitudinal prospective surveillance showed the annual incidence of 85.03 of ADL episodes per 1000 population in this rural Eastern Indian community. Similarly, a South Indian rural community, which is endemic for bancroftian filariasis recorded the annual incidence of 96.3 per 1000 population [6]. The incidence rates are available from a few other endemic countries such as Ghana (96 per 1000 population) [5] and Tanzania (33 per 1000 population) [8]. The proportion of people affected with ADL episodes in the present study (5.4%) is similar to that reported from another bancroftian filarial endemic community from South India (5.3%) [6]. The age wise distribution indicates that male recorded higher incidence in all age groups except in the age groups of 41–50 years and above 60 years of age. This age-wise incidence follows the pattern with the prevalence of microfilaraemia and chronic disease [13]. The variation could be due to differences in prevalence of chronic disease and differential susceptibility to ADL episodes for individuals with lymphoedema and hydrocele. In the present population, men recorded higher prevalence of chronic filarial disease than women [10]. The mean age of affected individuals is varying between male and female. The lower mean age of male patients may be due to occurrence of hydrocele even at lower age. In the present study population, majority of affected individuals has only one episode per year and only 27.8% of affected individuals have experience more than once. The data showed that these multiple episodes are more common in patients with chronic disease. The mean number of episodes per year is significantly higher among lymphoedema patients than even hydrocele patients. A study from South India reported a direct relationship between the number of acute attacks and the grade of lymphoedema [14]. It is also known that the frequency of these attacks is generally higher in bancroftian filariasis as compared to brugian filariasis [14,15]. Regarding the seasonal variation in the frequency of ADL episodes, it is lower in winter than is summer and rainy season. Some of the earlier studies reported higher frequency in rainy season [16,17]. In Tanzania, the higher incidence of ADL episodes during rainy season is related to increased transmission by infective mosquito bites [8]. It is consistent with the hypothesis that ADL episodes may be associated with allergic responses to massive parasite antigen release [18,19]. There is uniformity in the associated symptoms of ADL episodes amongst various endemic communities, though the etiology of the ADL is not clear. The majority of the ADL patients in this study are amicrofilaraemic, and this finding is in conformity with earlier observations [5,19,20]. The limitation of this study is that an insensitive method of microfilaria detection was used, i.e., finger prick method as opposed to Nucleopore filtration method.

The incidence and duration of the ADL episode has greater economic implication on individuals, their families and community. There will be substantial loss of work during these episodes and subsequent economic loss [21-23]. In the present study population, on average each episode persists for 4 days, which is similar to the other endemic areas [6,8,16,24,25]. The magnitude of loss due to ADL episodes is substantial and it will be constituted as a major proportion to the total burden of LF. While estimating the global burden of LF, only the chronic forms of disease were considered [26], perhaps due to lack of data at that time. As the data on incidence and duration of ADL episodes are available at least from some endemic areas, they should be considered during further estimation of disease burden due to LF. In the present study community, it is reported that the chronic condition is posing considering economic burden due to loss of work and treatment costs [27]. It is well known that the disability and economic loss caused by chronic filariasis is life long and much higher. And additional incapacity caused by the ADL episodes, majority of which occur in these chronic filarial patients, further poses the burden on the individuals and their families. It is clear that there are two types of ADL episodes, ADL secondary to bacterial or fungal infections and ADL caused directly by the parasite infection itself [1]. For the episodes among chronic lymphoedema cases, secondary bacterial infection may be plausible explanation [28-30]. It is also evident from recent findings that simple foot hygiene and prevention of secondary bacterial infection lower the incidence of ADL episodes [28,31-33]. Shenoy et al. [34] demonstrated that well designed programme of foot care significantly decreases the frequency of ADL episodes. In this programme, meticulous hygiene including use of foot wear, regular washing of affected limbs, etc. to prevent injuries and infections needs to be incorporated. Thus, it is essential to develop and promote simple, cost-effective and user-friendly measures to minimise the burden of acute disease of LF.

## Competing interests

The author(s) declare that they have no competing interests.

## Authors' contributions

BVB conceived and designed the study; performed analysis and interpretation of data; and drafted the paper. ANN collected and computerised the data; KD collected the data. All authors read and approved the final manuscript.

## Pre-publication history

The pre-publication history for this paper can be accessed here:


